# Developmental brain trajectories in children with ADHD and controls: a longitudinal neuroimaging study

**DOI:** 10.1186/s12888-016-0770-4

**Published:** 2016-03-11

**Authors:** Timothy J. Silk, Sila Genc, Vicki Anderson, Daryl Efron, Philip Hazell, Jan M. Nicholson, Michael Kean, Charles B. Malpas, Emma Sciberras

**Affiliations:** Murdoch Childrens Research Institute, Melbourne, Australia; The Royal Children’s Hospital, Melbourne, Australia; Department of Paediatrics, University of Melbourne, Melbourne, Australia; Discipline of Psychiatry, University of Sydney, Sydney, Australia; Judith Lumley Centre, La Trobe University, Melbourne, Australia; School of Psychology, Deakin University, Melbourne, Australia

**Keywords:** Attention deficit hyperactivity disorder, Neuroimaging, Longitudinal, Protocol, Trajectory, Outcomes

## Abstract

**Background:**

The symptom profile and neuropsychological functioning of individuals with Attention Deficit/Hyperactivity Disorder (ADHD), change as they enter adolescence. It is unclear whether variation in brain structure and function parallels these changes, and also whether deviations from typical brain development trajectories are associated with differential outcomes. This paper describes the Neuroimaging of the Children’s Attention Project (NICAP), a comprehensive longitudinal multimodal neuroimaging study. Primary aims are to determine how brain structure and function change with age in ADHD, and whether different trajectories of brain development are associated with variations in outcomes including diagnostic persistence, and academic, cognitive, social and mental health outcomes.

**Methods/Design:**

NICAP is a multimodal neuroimaging study in a community-based cohort of children with and without ADHD. Approximately 100 children with ADHD and 100 typically developing controls will be scanned at a mean age of 10 years (range; 9–11years) and will be re-scanned at two 18-month intervals (ages 11.5 and 13 years respectively). Assessments include a structured diagnostic interview, parent and teacher questionnaires, direct child cognitive/executive functioning assessment and magnetic resonance imaging (MRI). MRI acquisition techniques, collected at a single site, have been selected to provide optimized information concerning structural and functional brain development.

**Discussion:**

This study will allow us to address the primary aims by describing the neurobiological development of ADHD and elucidating brain features associated with differential clinical/behavioral outcomes. NICAP data will also be explored to assess the impact of sex, ADHD presentation, ADHD severity, comorbidities and medication use on brain development trajectories. Establishing which brain regions are associated with differential clinical outcomes, may allow us to improve predictions about the course of ADHD.

## Background

Attention-deficit/hyperactivity disorder (ADHD) is one of the most common neurodevelopmental disorders of childhood, affecting 5 % of school-age children [1]. ADHD has a major impact on everyday functioning, with affected children experiencing significant and lasting impairments across multiple domains including mental health, academic, cognitive, social, and family functioning [2–5]. Although 30–40 % of affected children show a reduction of symptoms in adolescence [6], related impairments are enduring [2, 3] and are associated with increased risk of poor academic achievement and early school dropout, increased rates of criminality, substance abuse and mental health disorders [7].

The Children’s Attention Project (CAP) [8] is an Australian longitudinal study of community-based children with ADHD and non-ADHD controls, mapping the developmental course of ADHD symptoms, and identifying risk and protective factors associated with differential outcomes. It is tracking an extremely well-phenotyped sample assessed at mean ages 7, 8.5, and 10 years. Data are collected on current ADHD status, comorbidities, key functional domains relevant to ADHD (mental health, cognitive, academic and social functioning), medication use, and general wellbeing.

This paper describes the Neuroimaging of the Children’s Attention Project (NICAP). This extension to CAP involves comprehensive longitudinal multimodal neuroimaging to determine how brain structure and function change over developmental stages in ADHD, and whether deviations from typical trajectories of brain development are associated with differential outcomes, such as the persistence or remission of ADHD symptoms and academic, cognitive, social and mental health outcomes. This paper describes the rationale, design and methodology of the neuroimaging protocol for NICAP.

### ADHD and the developing adolescent brain

As individuals enter adolescence, the presentation of some ADHD symptoms and neuropsychological functioning appears to change [9–12]. Inattentive symptoms remain relatively constant from ages 9–12 years, whereas hyperactive/impulsive symptoms decline although they do not normalize [13, 14]. The transition to adolescence is an important developmental shift with major environmental and biological changes potentially exerting influence on functional status. This includes the transition to high school which has been associated with an interruption in the decline in ADHD symptomotology [9]. It also corresponds with the major physiological and emotional effects of puberty.

Numerous cross-sectional studies have examined brain differences between individuals with and without ADHD. Functional imaging studies have highlighted several abnormalities in individuals with ADHD, particularly in the prefrontal cortex and striatum (fronto-striatal circuits) and the parietal cortex [15–19]. Structural imaging studies have reported ADHD-related anomalies in the prefrontal cortex, cerebellum, striatum and basal ganglia, corpus callosum, and the parietal cortex [20–27]. There is debate as to whether these differences represent specific brain abnormalities characteristic of ADHD, or a delay of normal development (i.e., a maturation lag) [28]. However, there have been marked inconsistencies in previous studies, attributable to the use of small, homogenous clinical samples with considerable between-study variation in subtypes, gender and age. Generalizability to the larger/wider population of children with ADHD is therefore limited.

While ADHD symptoms vary considerably with age and neuroimaging abnormalities have been described in children with ADHD, it is unclear whether the changes in brain structure and function parallel symptom changes. It is also unknown whether deviations from typical trajectories of brain development are associated with differential outcomes, such as the persistence or remission of ADHD symptoms and academic, cognitive, social and mental health outcomes. A recent meta-analysis [29], identified the need for longitudinal designs and larger samples to advance the field. For our understanding of the neural underpinnings of ADHD to progress and generate knowledge that will inform treatments, there is a need to establish working models of neurodevelopment. This can best be achieved by linking serial measures of brain development, using multiple state-of-the-art neuroimaging methods, with detailed phenotypic and functional outcomes indices.

NICAP will collect single site, multimodal neuroimaging on a high-resolution 3 Tesla scanner to link neurobiological structure and function to academic, cognitive, social, and mental health outcomes. We will assess children with and without ADHD as they progress through puberty at mean ages 10, 11.5 and 13 years. This design will enable us to map trajectories in brain growth and how they differ between typically developing children and those with ADHD. We will be able to ascertain whether such changes are reflected in ADHD symptomatology and functional abilities.

### Study aims

The primary aims are to 1) Describe how brain structure (whole-brain volume, grey-matter volume, white-matter volume, cortical thickness, diffusion indices) and function (resting state connectivity) change across late childhood to early adolescence (brain growth trajectories) for children with and without ADHD; and 2) Examine whether differences in trajectories of brain structure and function reflect differential outcomes for children with ADHD and non-ADHD controls. Outcomes to be assessed include the persistence of ADHD, ADHD symptom severity and functional outcomes (academic, cognitive, social, and mental health).

Secondary aims will explore the impact of sex, ADHD presentation, ADHD severity, comorbidities and medication use on brain growth trajectories.

## Methods/Design

NICAP is single site, multimodal neuroimaging study in a community-based cohort of children with and without ADHD conducted longitudinally over a 5-year period. Baseline, 18 and 36 month follow-ups will be conducted between 2014 and 2018. The study is funded by the Australian National Medical Health and Research Council (NHMRC; project grant #1065895). Ethics approval was granted by the Royal Children’s Hospital Human Research Ethics Committee, Melbourne (#34071).

### The cohort

#### CAP (2011–2015)

Participants for NICAP are recruited from the CAP. The CAP cohort and methods have been described previously [8]. Briefly, children were screened for ADHD using both parent and teacher reports on the Conners 3 [30] ADHD Index (*N* = 6098) in their second year of formal schooling. Surveys were distributed across 43 socio-economically diverse Melbourne primary schools. Children screened positive as potential ADHD cases (both the parent and teacher ADHD indices were ≥75th percentile for age for boys, and ≥80th percentile for girls) and a matched sample of those screened negative (both parent and teacher ADHD indices were <75th percentile for boys and <80th percentile for girls) received a parent face-to-face diagnostic interview to confirm diagnostic status. Baseline data were collected between 2011 and 2012, for a sample of 179 children with confirmed ADHD and 212 confirmed non-ADHD controls aged 7 years. Participants were followed up at two 18 month intervals at ages 8.5 and 10 years.

#### NICAP (2014–2018)

Recruitment for NICAP participation coincides with the CAP 36 month data collection (age 10 years). Parents provide additional written informed consent for the NICAP study. Approximately 100 ADHD and 100 typically developing controls will be recruited for NICAP baseline assessment. Two follow-up assessments will occur at 18-month intervals when participants are aged 11.5 and 13 years.

### Power and sample size

Our sample size is primarily based on the feasibility of recruitment from the pre-existing CAP cohort. Based on recruitment of equal numbers of controls to ADHD cases, with a participation rate of 65 %, accounting for exclusions due to MRI incompatibility (e.g., dental braces), and an estimated attrition of 5 % at each timepoint, we expect ~100, 95 and 90 participants per group at timepoints 1, 2 and 3 respectively. It is difficult to estimate with precision the power this will provide for detecting group differences in trajectories of development. However, a study of the sample size required in longitudinal MRI studies of brain volume in adults [31] suggests 80 % power detection of a 5 % difference between two groups for change in even small subcortical structures (e.g., the caudate) from a sample size of 90–104 per group.

### Procedure

At each data collection time-point participating families will attend a 3.5 h assessment session at The Royal Children’s Hospital, Melbourne, Australia. Assessment sessions involve a structured diagnostic interview, parent questionnaire, child cognitive assessment and MRI scanning. Saliva samples will also be collected for future research questions around genetics and pubertal hormones. With parent consent, questionnaires will be sent to the child’s classroom teacher.

Measures are summarized in Table 1. Children will be assessed in their usual classroom condition, therefore, if the child is currently using medication, they are not asked to cease medication for the assessment, but details of medication history and dosage are recorded. Research staff conducting assessments will be blinded to the child’s diagnostic status.

**Table 1:** Summary of assessment measures for NICAP

Measures		Source	Timepoint				
			CAP		NICAP		
			1	2	1	2	3
Diagnostic Interview							
ADHD & comorbidities	DISC-IV; structured clinical interview [32]	P	•		•		•
Magnetic Resonance Imaging							
	Structural T1	C			•	•	•
	Structural T2	C			•	•	•
	Multishell DWI	C			•	•	•
	Resting state fMRI	C			•	•	•
	Quantitative susceptibility mapping	C			•	•	•
Cognitive Assessment							
Intellectual functioning	WASI: vocabulary, matrix reasoning [52]	C	•		•	•	•
Language	CELF 4th edition: screening test [53]	C	•		•	•	•
Academic achievement	WRAT 4: word reading, numeracy [54]	C	•		•	•	•
Working memory	Computerised spatial n-back	C			•	•	•
Inhibition	Computerised Stop-signal task	C			•	•	•
Sustained attention	Computerised SART	C			•	•	•
Spatial attention	Landmark task	C			•	•	•
Cognitive flexibility	Computerised set-shifting task	C			•	•	•
Visual-motor	Grooved pegboard test	C			•	•	•
Questionnaires							
Puberty development	Pubertal development scale; Tanner stage charts	P			•	•	•
Child functioning							
ADHD symptoms	Conner’ 3 parent & teacher ADHD index [30]	P, T	•	•	•	•	•
Autism Spectrum Disorder	SCQ - Lifetime version [55]; SSIS: Autism spectrum scale [56]	P	•	•	•	•	•
Mental health & social functioning	SDQ: Total problems score, emotional, conduct, peer and inattention-hyperactivity scale [57]	P, T	•	•	•	•	•
Social Skills	SSIS: Responsibility, self-control, bullying, communication and engagement scales [56]	P, T	•	•	•	•	•
Prosocial behaviours	SDQ: Prosocial behaviour [57]	P, T	•	•	•	•	•
Victimisation	SEQ: Physical victimisation, relational victimisation [58]	P		•	•	•	•
Quality of Life	Pediatric quality of life inventory (Peds QL v4) [59]	P	•	•	•	•	•
Health	Medication history, child global health, sustained injuries, allied health services use	P	•	•	•	•	•
Home environment							
Parental mental health	Kessler 6 (K6): psychosocial symptom screener [60]	P	•	•	•	•	•
Family quality of life	CHQ: Emotional impact, time impact, family activities [61]	P	•	•	•	•	•
Family adversity	Stressful life events scale [62]	P	•	•	•	•	•
Parenting	LSAC parenting scales: parental warmth, hostility, consistency, parental self-efficacy [63]	P	•	•	•	•	•
Couple relationship	LSAC family functioning scales: parental conflict, support, and relationship satisfaction [63]	P	•	•	•	•	•
Pre/postnatal factors	LSAC prenatal & postnatal questions	P	•				
School environment							
Classroom performance	SSIS: Academic competence [56]	T	•	•	•		•
Teacher-child relationship	STRS (short form): conflict and closeness [64]	T	•	•	•		•
Teacher characteristics	Including teacher age, gender, teaching experience, education, self-efficacy, from LSAC; level of support.	T	•	•	•		•
Education services	Specialised school services, individual education plans, in-class assistance and grade repetition.	T	•	•	•		•
Physical Measures							
	Height, weight	C	•		•	•	•
	Saliva	C			•	•	•

Child ages for data collection timepoints are 7 years (CAP 1), 8.5 years (CAP 2) 10 year (NICAP 1), 11.5 years (NICAP 2) and 13 years (NICAP 3)

*Abbreviations*: *CELF* Clinical evaluation of language fundamentals, *CHQ* child health questionnaire, *DISC-IV* diagnostic interview schedule for children-IV, *DWI* diffusion weighted imaging, *fMRI* functional magnetic resonance imaging, *LSAC* longitudinal study of Australian children, *SART* sustained attention to response task, *SCQ* social comnmunication questionnaire *SDQ* strengths & difficulties questionnaire, *SEQ* social experience questionnaire, *SSIS* social skills improvement system; *STRS* student-teacher relationship scale, *TEA-CH* test of everyday attention for children, *WASI* Wechsler abbreviated scales of intelligence, *WISC* Wechsler intelligence scale for children, *WRAT* wide range achievement test

## Measures

### Diagnostic interview

*NIMH Diagnostic Interview Schedule for Children-IV* [32]: At baseline (10 years) and 36 months (13 years), parents complete the well-validated and widely used DISC-IV diagnostic interview (60–90 mins) to determine the participants’ ADHD status and comorbid mental health problems including anxiety, mood and externalizing disorders.

### Questionnaires

Questionnaires assess several domains covering a range of predictors and outcome variables. Key measures are described below and summarized in Table 1. Parent and teachers complete questionnaires pertaining to the child’s ADHD symptom severity and their social and emotional functioning. Parents also complete a series of questionnaires about the child’s functioning including emotional, physical, social and school quality of life, the child’s peer victimization, a screening measure for autism spectrum disorder symptoms, the child’s general health, the use of allied health services and medication history. Questionnaires concerning the home environment include a measure of family quality of life, stressful life events, and parents’ mental health. Numerous scales are drawn from the Longitudinal Study of Australian Children (LSAC; [33]) with items assessing parenting and the parent couple relationship. Retrospective questions regarding potentially relevant pre- and post-natal factors are also assessed, such as maternal alcohol use and smoking during pregnancy, gestational diabetes, preclampsia, stress/anxiety/depression/stressful life events during pregnancy, birth weight, gestational age, intensive care following birth and maternal postnatal depression. Teachers are asked questions around the child’s academic competence, the student-teacher relationship, as well as details on the teacher characteristics and education services. Those not described below have been described in detail elsewhere [8].

### Pubertal development

The Pubertal Development Scale (PDS) [34] is a parent-reported measure assessing development on five indices of pubertal growth. Parents are asked about whether the child’s secondary sexual characteristics have 1) not yet started, 2) barely started, 3) definitely started, 4) seems complete. Parents of males are asked about changes to voice and growth of facial hair and parents of females are asked about breast development and about the onset and age of menstruation.

The Tanner Sexual Maturation Scale (SMS) is a parent-reported measure used to assess the child’s pubertal stage. It comprises drawings of five progressive stages of pubertal development of secondary sexual characteristics, from stage 1 (pre-adolescent) through to stage 5 (adult appearance). For males, five drawings combining pubic hair and genital development are presented. For females, breast development and pubic hair development are presented in different drawings. Tanner staging has historically been considered the gold standard for puberty measurement [35].

### Cognitive assessment

NICAP includes direct assessments of working memory, inhibition, sustained attention, cognitive flexibility, spatial attention and visuo-motor integration. The first four tasks are computer-based, enabling measurement of properties such as reaction time.

The Stop Signal Task assesses response inhibition [36]. Subjects perform a choice reaction task and on a random selection of the trials, an auditory stop signal instructs subjects to withhold their response.

The Sustained Attention to Response Task (SART) is a measure of sustained attention [37]. The fixed version of SART is a repeating sequence of digits (1–9). Using a button press, participants respond to every digit (go-trial) except ‘3’ (no-go trial).

The Spatial N-Back is a widely used measure of working memory that requires flexible updating capabilities. This includes a spatial 1-back and 2-back version. The 1-back requires maintaining and updating one location at a time, whereas the more difficult 2-back requires maintaining and updating two locations.

The Set Shifting task assesses cognitive flexibility. Two target pictures are presented that vary along two dimensions (e.g., shape and color). Participants are cued with a letter to respond to the target pictures, according to one dimension.

The Landmark Task is a paper-based task measuring spatial attention [38]. Participants are presented with 20 examples of a bisected line, half are bisected exactly in the middle, while the remainder are bisected slightly offset to the left or right. Participants indicate which side of the line is shorter. Leftward or rightward spatial biases can be ascertained.

The Grooved Pegboard test (Lafayette Instruments, Lafayette, IN) is a timed motor test to assess complex visual-motor coordination for both the dominant and non-dominant hand. Participants place grooved pegs into a pegboard unit in an ordered pattern of 25 holes, requiring the participant to match the groove of the peg with the groove of the board.

### Neuroimaging procedure

#### Mock scanner training

Children complete a 30 min training session in a mock MRI scanner which reproduces the physical environment of the real scanner including sound recording of the scanner noises. This familiarizes participants to the MRI environment, lowers anxiety and provides practice at keeping still during the scanning session.

#### MRI scan

**Fig. 1:**
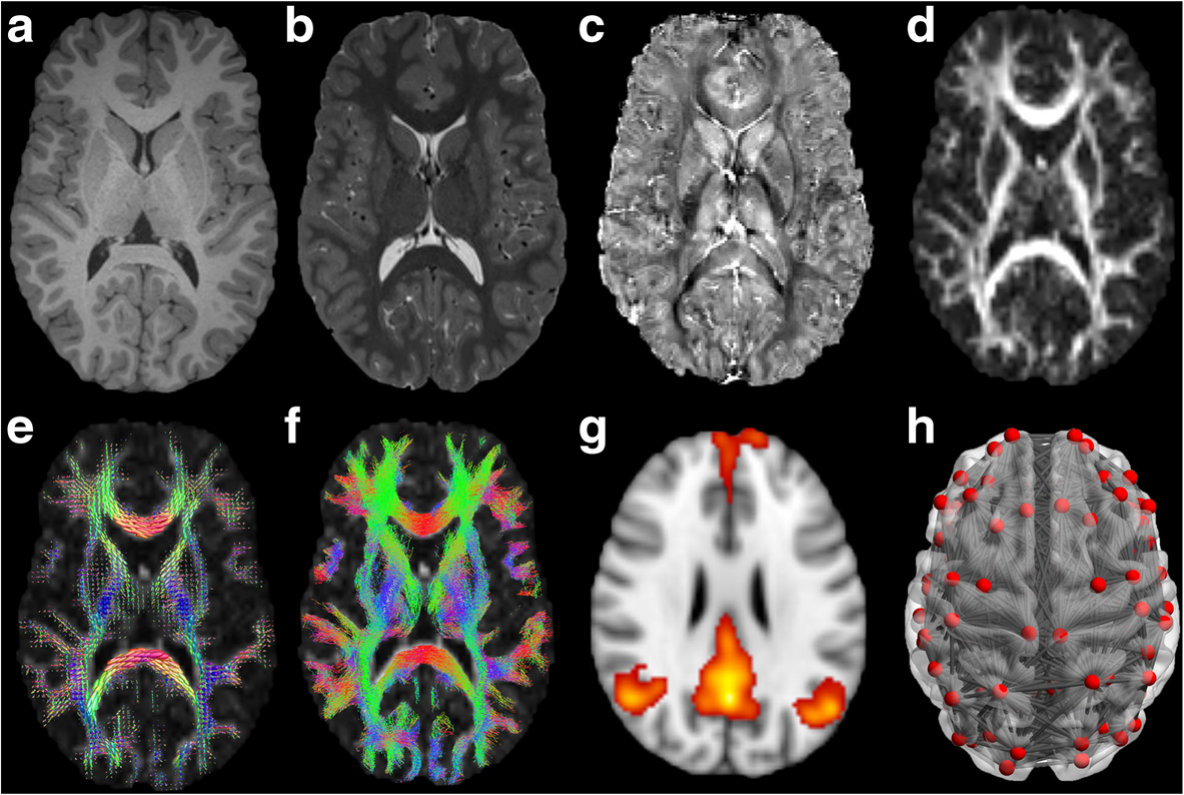
An example of the different sequences acquired to evaluate structural and functional development (**a**) T1-weighted; (**b**) T2-weighted; (**c**) Quantitative Susceptibility Mapping; (**d**) Diffusion Weight Imaging: fractional anisotropy (FA) map; (**e**) Diffusion Weight Imaging: estimation of the fibre orientation distribution; (**f**) Diffusion Weight Imaging: whole brain tractography; (**g**) resting state fMRI showing default mode network; (**h**) connectivity network for structural and function connectivity

Neuroimaging data are collected from a single-site on a research-dedicated 3-Tesla Siemens TIM Trio MRI scanner (Siemens, Erlangen, Germany) at the Murdoch Childrens Research Institute, The Royal Children’s Hospital, Melbourne. Using a 32-channel head coil, the multimodal MRI acquisition techniques have been selected to provide advanced information concerning the structural and functional development of the brain and regional development of specific structures. The neuroimaging protocol comprises structural and functional sequences lasting approximately 45mins. See Fig. 1 and Table 2 for sequence details.

**Table 2:** MRI sequence parameters for scanning

Sequence	T1w	T2w	DWI				fMRI		QSM
Type	MEMPRAGE	T2-SPACE	Shell 1	Shell 2	Shell 3	Blip Up/Down	rs-fMRI	Blip Up/Down	Multi-echo
TR (ms)	2530	3200	3200	3200	3200	3200	1500	3980	52
TE (ms)	1.77, 3.51 5.32, 7.2	532	110	110	110	110	33	33	7.38, 14.76, 22.14, 29.52, 36.90, 44.27
TI (ms)	1260	-	-	-	-	-	-	-	-
Flip angle (deg)	7	-	90	90	90	90	85	85	15
Slices	176	176	63	63	63	63	60	60	-
Voxel size (mm^3^)	0.9	0.9	2.4	2.4	2.4	2.4	2.5	2.5	1.0
FoV read (mm)	230	240	260	260	260	260	255	255	256
FoV phase (%)	90.6	89.8	100	100	100	100	100	100	68.8
Matrix	256 × 232	256 × 230	110 × 110	110 × 110	110 × 110	110 × 110	104 × 104	104 × 104	256 × 176
Band width (Hz/Px)	723, 751, 651, 651	610	1748	1748	1748	1748	1718	1718	210, 210, 210, 210, 210, 210
Echo spacing (ms)	10.1	3.76	0.69	0.69	0.69	0.69	0.69	0.69	-
Orientation	S	S	T	T	T	T	T	T	T
B value (s/mm^2^)	-	-	2800	2000	1000	0	-	-	-
No. directions/b = 0 s	-	-	60/4	45/6	25/6	-/2	-	-	-
Multi-band factor	-	-	3	3	3	3	3	1	-
Acquisition time	6 m 52 s	4 m 8 s	3 m 57 s	3 m 15 s	2 m 11	35 s (x 2)	6 m 33 s	24 s (x 2)	8 m 43 s

*S* sagittal, *T* transversal

#### Structural imaging

A modified multi-echo magnetization prepared rapid gradient-echo (MEMPRAGE) sequence, incorporating navigator based prospective motion correction, will be acquired to provide T1-weighted anatomical images [39, 40]. The MEMPRAGE sequence has many of the properties of a traditional MPRAGE sequence of distinguishing grey matter and white matter morphometry. The sequence averages multiple high bandwidth acquisitions reducing susceptibility artifacts and improving the contrast of the dura and subcortical structures, allowing for more accurate tissue segmentation.

We also employ Siemens in-scanner motion correction (MoCo) in which the field-of-view/slice positioning is updated in real time to accommodate for motion during the acquisition. This reduces motion artifact and dramatically improves image quality. This is particularly important in this population of children with attentional and hyperactivity difficulties, as motion artifact is a large challenge.

Additional morphometric information is obtained by employing the T2-SPACE (Sampling Perfection with Application optimized Contrast with flip angle Evolution) protocol to provide T2-weighted anatomical images. Together, the MEMPRAGE and T2-SPACE provides T1-weighted and T2-weighted volumes providing optimal sensitivity for tracking subtle changes in cortical morphometry.

#### Multi-band, multi-shell diffusion MRI

Diffusion-weighted images (DWI) are acquired to probe white matter microstructure. Multi-band accelerated EPI sequences protocol, developed by the Centre for Magnetic Resonance Research (CMRR, University of Minnesota), are acquired in order to accelerate DWI volume coverage allowing multiple shell acquisition. Three shells are acquired using this protocol (b = 2800, 2000, 1000 s/mm^2^ + interleaved b = 0 s/mm^2^) with an anterior-posterior phase encoding direction. Standard and reverse phase encoded blipped image with no diffusion weighting (Blip Up and Blip Down) are also acquired to correct for magnetic susceptibility-induced distortions related to the EPI acquisitions [41, 42].

Images are acquired with a multi-band acceleration factor of three. The advantage of using multi-band accelerated imaging is the reduced acquisition time, which allows the collection of multiple diffusion weightings (see Table 2) in the time it takes to collect just one typical diffusion weighting without multi-band acceleration. By acquiring three diffusion-weighted shells, we can obtain high angular resolution diffusion imaging (HARDI) required for spherical deconvolution tractography, as well as high signal-to-noise ratio (SNR) data for reliably assessing quantitative scalar metrics in white matter microstructure.

The DW processing pipeline uses a combination of purpose built neuroimaging tools from the MRtrix [43] and FSL [44] packages. First, raw images are corrected for susceptibility-induced geometric distortions, eddy current distortions, and inter-volume subject motion using EDDY and TOPUP toolboxes [45]. Corrected images then have all non-brain material “stripped” away by the BET tool [46]. For low b-value images, the diffusion tensors are calculated and scalar maps generated. For high b-value data, the images are prepared for constrained spherical deconvolution (CSD) tractography by: the estimation of a response function; estimation of the fibre orientation distribution (FOD); anatomically constrained tractography (ACT) in the white matter [47]; and SIFT2 to reconstruct streamline densities that are proportional to the fibre densities [48].

#### Multi-band resting state functional MRI

Resting state fMRI (rs-fMRI) images are acquired to measure spontaneous intrinsic correlated neural activity while subjects are at rest, enabling detection of functional connectivity between brain regions. rs-fMRI has longitudinal reliability and reproducibility in children [49], has the advantaged of not relying on task compliance, and avoids issues of age-appropriateness of task in longitudinal studies. Participants are instructed to keep eyes open and to look at a fixation cross. The multi-band accelerated EPI sequences (MB3), acquired as above, allows for 250 volumes with whole brain coverage to be acquired in a 6 min 33 s sequence.

The rs-fMRI processing pipeline begins with realignment of EPI volumes to correct for participant movement. Volumes are then aligned to the participant’s structural images, which are segmented into different tissue classes. Removal of physiological noise and other nuisance variables is then performed using a component-based approach. Signal from white matter and cerebrospinal fluid is used to estimate noise of non-neuronal origin (e.g., cardiac, respiratory). This noise is then removed from regions of interest, along with the contribution of realignment parameters and movement outliers.

#### Quantitative susceptibility mapping (QSM)

QSM provides a quantitative and spatially specific image contrast, which is differentially sensitive to myelin and iron content [50]. The novel sequence used in this study is a multi-echo spoiled-gradient-recalled (SPGR) sequence. We have optimized the acquisition protocol to reduce acquisition time to 8 min and 43 s, which is more feasible for a pediatric population. The QSM and phase reconstruction algorithm [51] are employed to process and analyze the data.

### Quality control

Quality control procedures are important at a number of steps. Regarding MRI motion artifacts, several steps are taken in order to minimize movement during the scanning and to assess the data quality afterwards. The mock scanner session prior to the scan is vital to assist participants be aware of movements and become comfortable in the scanner environment. During all scans except for the rs-fMRI participants watch a movie of their choice distracting them from the scanning environment. During scanning, movement is monitored and participants are reminded to keep still when necessary. Poor images due to motion are repeated if time permits. At the scanner level, scanner stability is monitored weekly with the standard functional Brain Imaging Research Network (FBIRN) QA protocol. In addition, T1 and T2 are performed to examine signal-to-noise and image uniformity.

### Staff training and supervision

Research staff and students who conduct the assessments are trained to a high level of competence in the scanning and assessment procedures, and are observed for the first two assessments. Written standard operation procedures were developed for standardized assessments, cognitive testing, mock scan and saliva collection. Fortnightly supervision meetings take place with a registered clinical psychologist (ES) in order to maintain consistency across cognitive and diagnostic assessments.

## Discussion

This research will provide the ability to map trajectories of brain structure and function onto a comprehensive set of functional outcome domains encompassing academic, cognitive, social, and mental health functioning. Developing a large database of multimodal MRI sequences with ongoing clinical and cognitive/behavioral measures in a demographically diverse sample will enable the detection of subtle, yet important, differences in brain developmental trajectories in children with ADHD compared to non-ADHD peers.

Identifying objective neural markers of outcomes in ADHD, and potential modifiable predictors of outcomes will be an important innovation and will contribute substantially to improving the prognosis of children with ADHD. Establishing which brain regions are associated with positive clinical outcomes will help improve predictions about the course of ADHD. The advantage of a large community sample is the opportunity to examine neurobiological development across the continuum of severity, as well as in healthy controls.

A better understanding of the developmental links between brain changes and outcomes also has important implications for children with developmental and mental health problems broader than ADHD. The identification of neurodevelopmental changes associated with functional outcomes will open the possibility for future studies to test targeted interventions leading to improved long-term outcomes.
